# Validation and translation of the Hungarian version of the Female Sexual Function Index (FSFI-H)

**DOI:** 10.1007/s00192-019-04049-x

**Published:** 2019-07-29

**Authors:** Márta Hock, Nelli Farkas, István Tiringer, Stefánia Gitta, Zoltán Németh, Bálint Farkas

**Affiliations:** 1 Institute of Physiotherapy and Sport Sciences, Faculty of Health Sciences, University of Pécs, Pécs, Hungary; 2 University of Pécs, Institute of Bioanalysis, Pécs, Hungary; 3 Institute of Behavioral Sciences, Medical School University of Pécs, Pécs, Hungary; 4 Department of Gynecology, Hospital St. John of God, Vienna, Austria; 5 Member of the MTA-PTE Human Reproduction Scientific Research Group, Hungarian Academy of Sciences (MTA), Budapest, Hungary; 6 Department of Obstetrics and Gynecology, University of Pécs, 17 Édesanyák str., Pécs, H-7624 Hungary

**Keywords:** FSFI, FSFI-H, Hungarian language adaptation, Sexual functioning, Validation

## Abstract

**Introduction and hypothesis:**

The Female Sexual Function Index (FSFI) has been used for clinical and research purposes in many countries. The aim of this study was to translate, adapt and perform a psychometric validation of a Hungarian version of the FSFI.

**Methods:**

The FSFI was translated into Hungarian, and its precision was ascertained through reverse translation by an expert team. As a first step, 40 volunteers participated in an evaluation of the test-retest reliability of the Hungarian version over a 2-week period. After that, 418 (331 control and 87 with pelvic organ prolapse) women who had been in a stable sexual relationship in the previous 4 weeks participated in the study. The data were summarized using descriptive statistics. The structure validity was examined by confirmatory factor analysis, with which we tested the hypothesized original factor structure, using maximum likelihood model estimation. We calculated the Comparative Fit Index (CFI), root mean square error of approximation (RMSEA), standardized root mean residual and Akaike information criterion (AIC). To test the internal consistency, Cronbach’s alpha coefficients of the full scale were determined. Spearman’s rank correlation was used for testing divergent validity and Mann-Whitney U-test for examining discriminant validity.

**Results:**

The FSFI was easily understandable and acceptable as well as capable of adequately evaluating and measuring various aspects of female sexual functioning. A high degree of internal consistency was demonstrated by the Cronbach’s alpha value (0.963).

**Conclusion:**

The FSFI Hungarian version is a valid tool that measures the same functioning as the original English questionnaire.

## Introduction

Several benign and malignant gynecologic diseases have negative impacts on different segments of life, which may lead to sexual dysfunction. Sexual dysfunction can arise from other physical, social and psychologic factors as well [1]. These may cause problems during any phase of the sexual response cycle, preventing the individual from experiencing satisfaction during sexual activity. The prevalence of female sexual dysfunction (FSD) varies worldwide between 8 and 75% [2–7]. In Hungary, only a small number of women seek professional help. These patients usually meet gynecologists as a primary physician, and then their treatment includes psychologists or sexologists; therefore, outpatient gynecologic counseling provides an important setting for conducting research, diagnosis and treatment of FSD [8]. The Female Sexual Function Index (FSFI) was designed to evaluate sexual function in a general population. The translation and validation of the FSFI allow the questionnaire to be applied in daily clinical practice, for research purposes and to estimate women’s sexual function. The aim of the study was to adapt the FSFI questionnaire and to assess the reliability and validity of the Hungarian version among sexually active women.

## Materials and methods

Women aged between 18 and 77, who had been sexually active and in a stable relationship for at least 4 weeks prior to the study, were recruited as volunteers in the current cross-sectional study design. The majority of the participants had high school or university diplomas. Participants (*n* = 331) were healthy university students (Institute of Physiotherapy and Sport Sciences, Faculty of Health Sciences, University of Pécs). After a short personal interview focusing on the general health conditions and medical history (carried out by MH and SG), the participants filled out online questionnaires. The exclusion criteria were concurrent sexually transmitted diseases (STD), prior or current malignancy, neurologic and psychiatric diseases (depression, schizophrenia, mental disabilities), severe somatic diseases, e.g., thyroid dysfunction, liver dysfunction, unstable coronary heart disease, addiction to psychoactive substances and/or alcohol, use of medications affecting sexual function (antipsychotics, antidepressants, antihistamines, benzodiazepines), pregnancy or being within 3 months postpartum, and illiteracy. The subjects were informed that their participation in the survey was anonymous and completely voluntary. This study was approved by the Institutional Review Board of the Faculty of Medicine, University of Pécs, in 2017 (no. 6920).

### FSFI

The FSFI is a 19-item self-report questionnaire of female sexual functioning [9] consisting of six dimensions: desire (Q1, 2), arousal (Q3, 4, 5, 6), lubrication (Q7, 8, 9, 10), orgasm (Q11, 12, 13), satisfaction (Q14, 15, 16) and pain (Q17, 18, 19). The FSFI assesses sexual functioning over the past 4 weeks. The subscale scores range from 1 to 5 for items 1, 2, 15 and 16. For all other items, the range was from 0 to 5 with the supplementary option “no sexual activity.” The full-scale score ranges from 2.0 to 36.0, where the higher score is associated with less severity of sexual dysfunction. The questionnaire showed a high degree of internal consistency (Cronbach’s α values ≥ 0.82) and high test-retest reliability for each domain (Spearman’s rho = 0.79–0.86). It has been successfully cross-validated, and a diagnostic cutoff score of 26.55 has been determined for classification of total FSD [10].

### Short form 36 (SF-36) survey

The SF-36 measures health-related quality of life (QoL) across eight domains, which can be summarized as physical and mental health. The eight domains are as follows: physical functioning (10 items; Cronbach’s α 0.93); role limitations due to physical health problems (4 items, Cronbach’s α 0.84); role limitations due to emotional problems (3 items; Cronbach’s α 0.83); energy and vitality (4 items; Cronbach’s α 0.86); mental health (5 items; Cronbach’s α 0.90); social functioning (2 items; Cronbach’s α 0.85); bodily pain (2 items; Cronbach’s α 0.78); general health (5 items; Cronbach’s α 0.78). These scores are transformed into a domain score ranging from 0 to 100, with a higher score representing higher levels of health-related QoL [11, 12]. The four domain scores are averaged to a physical component score (PCS), and four other domain scores are averaged to a mental component score (MCS). An additional item with health change is not integrated into the PCS or MCS scores [13]. The Hungarian adaptation of the questionnaire and the determination of normal values were carried out by Czimbalmos et al. [14]. The SF-36 was completed at the same time as the FSFI-H for comparison.

### Translation of the FSFI into Hungarian (FSFI-H)

The linguistic validation was carried out in accordance with the guidelines of linguistic validation processes [15]. The original FSFI was translated from English into Hungarian by two physicians, who are fluent in both English and Hungarian (version 1). The back translation of the FSFI-H into the original language was carried out by an independent bilingual investigator and was reviewed by the author to obtain a reliable translation (version 2). The translation was further reviewed by a ten-member expert committee, including gynecology specialists, PhD students, health sciences university teachers and behavioral science specialists, to achieve a reliable Hungarian version of the FSFI questionnaire (version 3). Then, a face-to-face interview was also conducted with women to check for any difficulties in understanding and interpreting the questions. No major difficulties were noted.

### Divergent validity

Since no validated Hungarian questionnaires that measure the same context as the FSFI are available, the SF-36 questionnaire, a self-evaluated global quality-of-life measure, was applied as a benchmark.

### Discriminant validity

To examine whether the Hungarian version of the FSFI can detect differences between a general/healthy population and a clinical population, we used retrospective data of a group of women with pelvic organ prolapse (POP), which is known to be correlated with decreased sexual quality of life because of the altered genital anatomy, decreased lubrication, and involuntary intracoital urine or fecal incontinence [16]. Patients (*n* = 87) were diagnosed in a urogynecologic outpatient clinic (Győr, Hungary). All patients provided written informed consent and volunteered to be included. All women had ≥ stage 2 POP of the anterior, middle or posterior compartment, or a combination of them. All reported a sensation of a bulge in the vagina with or without symptoms of urinary, bowel or sexual dysfunction. (All methods, definitions and units conform to the standards set by the International Urogynecological Association and the International Continence Society, except where specifically noted [17].)

### Test-retest reliability

The final approved version was pretested in a pilot study on 40 women who were sexually active in a stable relationship 6 months prior to the study. The participants completed the same questionnaire twice with a 2-week interval. During the first visit, we conducted a face-to-face interview and collected demographic data. Finally, the test-retest correlations were evaluated by applying Spearman’s rank correlations and Bland-Altman plots between individual domains as well as in the full scales of the FSFI-H.

### Statistical analysis

**Fig. 1:**
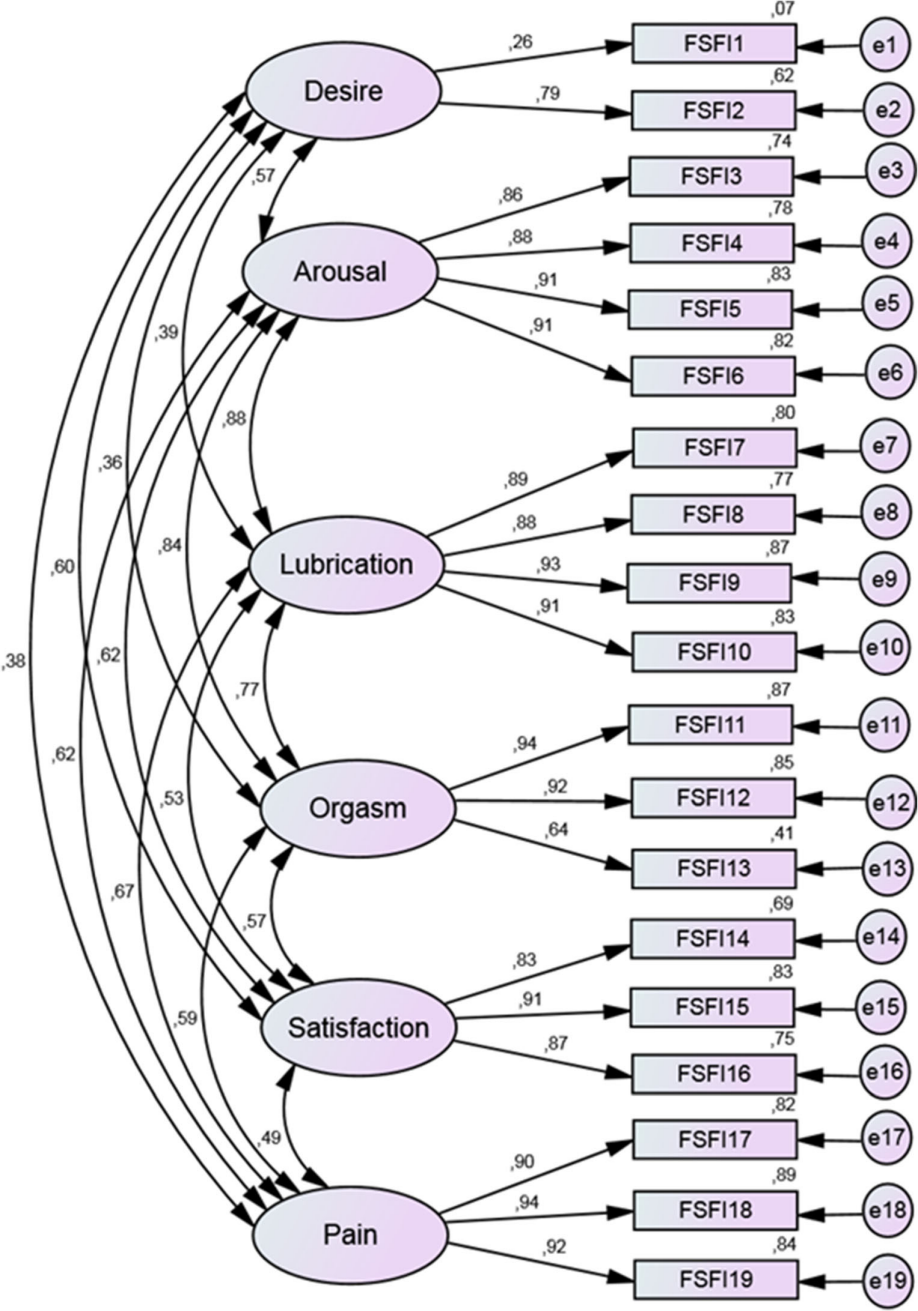
The six-factor model with factor loadings, correlations between factors and error terms

The data were summarized using descriptive statistics. The structure validity was examined by confirmatory factor analysis (CFA), with which we tested the hypothesized original factor structure, using maximum likelihood model estimation (Fig. 1). To determine which model had a better fit to our data, we compared generally offered fit indexes [Comparative Fit Index (CFI), root mean square error of approximation (RMSEA) and standardized root mean residual (SRMR)]. Acceptable values of the CFI are ⋝ 0.95, of the RMSEA ⋜ 0.08 and of the SRMR ⋜ 0.11 [18]. Additionally, on the basis of the Akaike information criterion (AIC), the six- and five-factor structures were compared considering which had a better fit to our data set [19]. To test the internal consistency, Cronbach’s alpha coefficients were determined. Spearman’s rank correlation was used for testing divergent validity and the Mann-Whitney U-test for examining discriminant validity [20]. CFA was performed using AMOS (version 5); all other analyses were carried out with the IBM-SPSS version 25 software package. The results were considered significant if *p* < 0.05.

## Results

A total of 418 women were enrolled in the current study; 331 constituted the general/control group, with a mean age of 33.58 ± 8.94 years. (The majority, 47% of the participants, had high school or university diplomas, and 71.9% lived in urban areas.)

Eighty-seven women with pelvic organ prolapse (POP) comprised the clinical group, with a mean age of 47.86 ± 12.27 years (POP stage II to IV, 64.5%, 17.5% and 1%, respectively), which was used for the discriminant validity.

### Structure validity

Confirmatory factor analysis is a tool to verify a given theoretically based factor structure. Previous studies showed either a six- or five-factor structure in the case of FSFI: desire, arousal, orgasm, lubrication, pain, satisfaction (desire and arousal constitute a unique factor in the five-factor model) [21]. We analyzed the five- and six-factor models, and the six-factor model showed an acceptable [CFI = 0.957, RMSEA = 0.76 (CI: 0.66–0.85), SRMR = 0.514, AIC = 439.1] and superior fit compared with the five-factor model [CFI = 0.949, RMSEA = 0.81 (CI: 0.72-0.09), SRMR = 0.57, AIC = 505.1]. All factors except desire had acceptable standardized regression weights (range 0.644–0.942). The answers to the first item about the frequency of desire were different from the answers given to the second item about the level of desire. Spearman’s rho between item 1 and 2 was only 0.187 (*p* = 0.001). Correlations among factors were high (0.484–0.872).

### Reliability

Cronbach’s α coefficients were determined for the total and domain scores of the FSFI-H, which were high. In the subscales, they ranged from 0.423 to 0.981. The coefficient was 0.963 for the full scale. The total FSFI domain scores obtained from the enrolled women are presented in Table 1.

**Table 1:** Mean values and Cronbach’s α coefficients of the FSFI-H

Domains	Score range	Factor	Mean ± SD	Cronbach’s alpha	Number of items
Desire	1.2–6.0^a^	0.6	3.60 ± 1.01	0.423	2
1. Frequency	1.0–5.0		3.10 ± 1.08		
2. Level	1.0–5.0		3.04 ± 0.97		
Arousal	0.0–6.0^a^	0.3	4.02 ± 1.946	0.973	4
3. Frequency	0.0–5.0		3.62 ± 1.73		
4. Level	0.0–5.0		3.19 ± 1.50		
5. Confidence	0.0–5.0		3.40 ± 1.61		
6. Satisfaction	0.0–5.0		3.58 ± 1.72		
Lubrication	0.0–6.0^a^	0.3	4.60 ± 2.11	0.981	4
7. Frequency	0.0–5.0		3.85 ± 1.78		
8. Difficulty	0.0–5.0		3.95 ± 1.76		
9. Frequency of maintaining	0.0–5.0		3.86 ± 1.73		
10. Difficulty in maintaining	0.0–5.0		4.05 ± 1.75		
Orgasm	0.0–6.0^a^	0.4	4.28 ± 2.03	0.898	3
11. Frequency	0.0–5.0		3.65 ± 1.78		
12. Difficulty	0.0–5.0		3.69 ± 1.80		
13. Satisfaction	0.0–5.0		4.18 ± 1.44		
Satisfaction	1.2–6.0^a^	0.4	4.28 ± 1.46	0.931	3
14. With closeness with partner	1.0–5.0		4.33 ± 1.29		
15. With sexual relationship	1.0–5.0		4.27 ± 1.34		
16. With overall sex life	1.0–5.0		3.98 ± 1.45		
Pain	0.0–6.0^a^	0.4	4.46 ± 2.12	0.971	3
17. Frequency during vaginal penetration	0.0–5.0		3.77 ± 1.79		
18. Frequency following vaginal penetration	0.0–5.0		3.79 ± 1.78		
19. Level during or following vaginal penetration	0.0–5.0		3.86 ± 1.72		
Full-scale score	5.2–36.0^b^		25.25 ± 9.27	0.963	19

*SD* standard deviation

^a^The individual domain scores were calculated by adding the scores of the individual items that comprise the domain and multiplying the sum by the domain factor

^b^The full-scale score is calculated by adding the six domain scores

### Divergent validity

No significant correlations between the results of the FSFI-H and SF-36 were found with Spearman’s rank correlation analyses among either the subscales or the total scores.

### Discriminant validity

To establish differences between the control and POP patient group, the total and subscale scores of both groups were compared. In the analysis we used an age-matched group (45 ± 5 years) from the original general control group. The results showed significant differences in the case of arousal, lubrication, orgasm, satisfaction and the total score. (Table 2).

**Table 2:** Discriminant validity of the FSFI-H

	General	POP	*P* value
	Mean ± SD	Mean ± SD	
Desire	3.604 ± 1.007	3.889 ± 1.021	0.087
Arousal	4.015 ± 1.947	2.868 ± 1.023	0.001
Lubrication	4.605 ± 2.118	1.976 ± 0.981	0.001
Orgasm	4.276 ± 2.037	2.612 ± 1.24	0.001
Satisfaction	4.279 ± 1.459	2.627 ± 1.271	0.001
Pain	4.468 ± 2.13	4.808 ± 1.449	0.887
FSFI	25.246 ± 9.268	16.155 ± 3.449	< 0.01

Data were calculated with Mann-Whitney U-test

*POP* Pelvic organ Prolapse, *SD* standard deviation

### Test-retest reliability

**Fig. 2:**
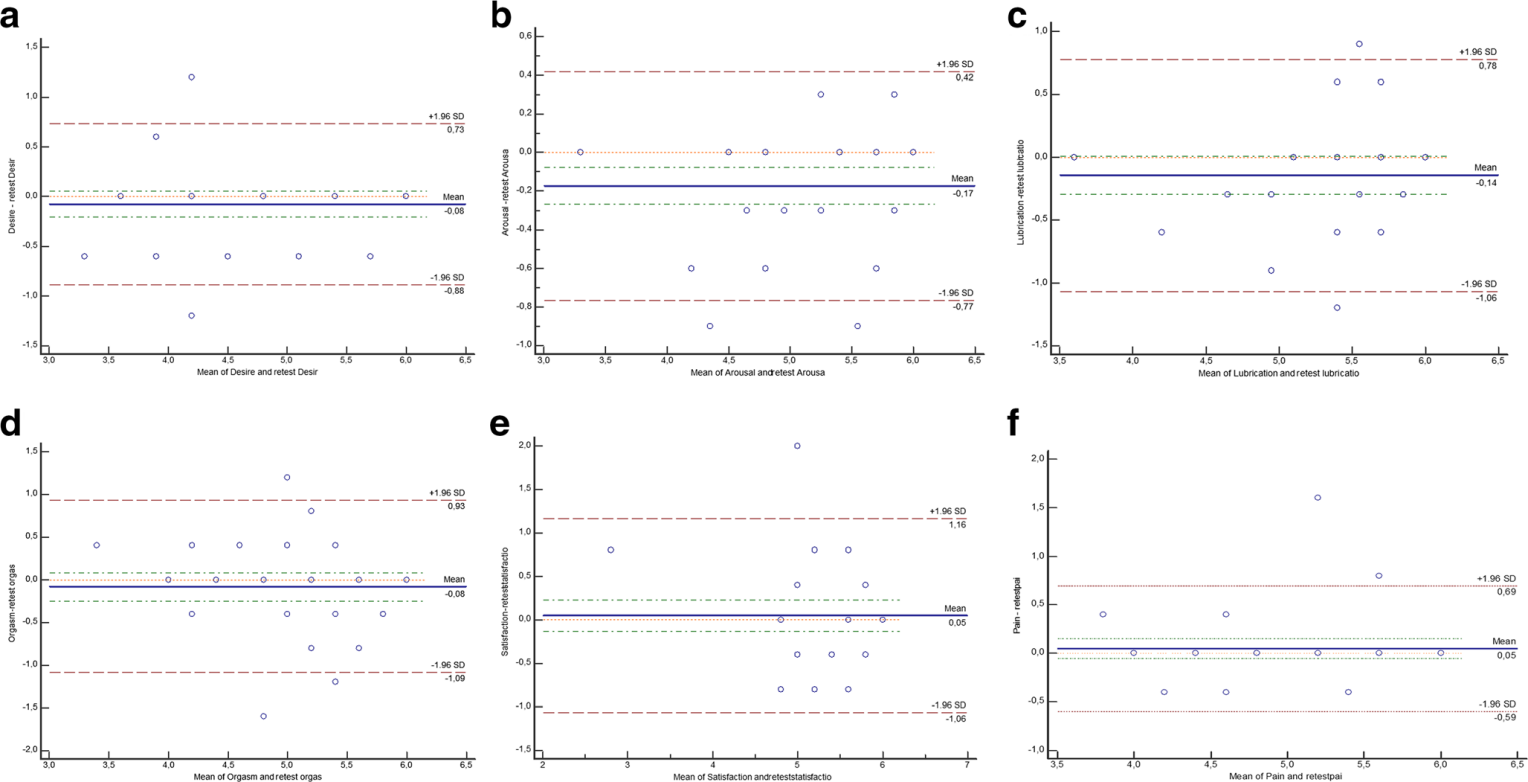
To compare two clinical measurements (test-retest), we used Bland-Altman plots. The mean values ranged between −0.17 and 0.05, and in all domains values fluctuated randomly between mean ± 1.96 SD, where (**a**) represents desire, (**b**) arousal, (**c**) lubrication, (**d**) orgasm, (**e**) satisfaction and (**f**) pain subscales

To compare two clinical measurements, we applied Bland-Altman plots. The values randomly fluctuated around the mean difference ± 1.96 SD (Fig. 2) in all domains. The test-retest reliability also showed strong and significant correlations between the domain and the full-scale scores (Spearman’s rho 0.490–0.903, *p* < 0.001) (Table 3).

**Table 3:** Results of the FSFI-H test-retest reliability

Domain	Phase	Mean	SD	Spearman rank correlation	*P* value
Desire	Test	4.485	0.744	0.851	< 0.001
	Retest	4.560	0.778		
Arousal	Test	5.063	0.656	0.884	< 0.001
	Retest	5.235	0.596		
Lubrication	Test	5.490	0.627	0.540	< 0.001
	Retest	5.633	0.534		
Orgasm	Test	5.190	0.669	0.704	< 0.001
	Retest	5.270	0.730		
Satisfaction	Test	5.530	0.579	0.490	0.001
	Retest	5.480	0.709		
Pain	Test	4.950	0.767	0.903	< 0.001
	Retest	4.900	0.751		
Total scale	Test	30.708	2.185	0.848	< 0.001
	Retest	31.078	2.248		

## Discussion

The FSFI is one of the most widely used and reliable questionnaires regarding sexual functioning in the female population. It has been translated and validated in about 30 languages and is used to assess FSD in women with different medical conditions, including vulvodynia, chronic pelvic pain, vulvar intraepithelial neoplasia, breast cancer and urinary incontinence [21–25]. The present study results demonstrated a very high internal consistency since the Cronbach’s alpha was 0.963 in the total scale. These high correlations are comparable to those reported by Rosen et al. in 2000 (≥ 0.82). Furthermore, we also found a high level of test-retest reliability with a Pearson product-moment correlation coefficient of 0.992 in the control population. During the test-retest reliability analysis, the translation was found to be very appropriate; the test-retest reliability showed significant correlation between domains and full-scale scores. Our CFA results showed an acceptable fit to the six-factor model frequently found in previous validation studies. All fit indices were in the acceptable range, indicating that the FSFI-H can measure the same domains as the original questionnaire. By collecting data from over 300 participants, we managed to examine both pre- and postmenopausal women. The applicability of the desire factor needs to be examined in a further study because the present study did not exclude participants who had difficulties with the desire caused by sexually related personal distress, hypoactive desire disorders, etc., due to the lack of sexologists in the research group. Nevertheless, we must also consider the possibility of cultural differences [26, 27]. Based on the results of the discriminant validity analysis, which included women with pelvic organ prolapse and demonstrated considerably lower total scores compared with healthy participants, it is assumed that the FSFI-H would be applicable to detecting sexual dysfunction as well.

## Strengths and limitation

The strength of our study was the relatively high number of participants and the high Cronbach’s alpha values. However, this study has several limitations. First, our study population failed to represent the average Hungarian female population, since the majority of the respondents were highly educated women living exclusively in urban areas. Our assumption is that this may have an effect on the respondents of the current study, who might be more open toward sexual issues compared with the average Hungarian woman. A further limitation of the study is that no concurrent validity was applied to obtain the cutoff point of the FSFI-H, unlike in other studies [28]. Finally, the results have a good statistical quality for confirming the linguistic validation, but they cannot fully confirm the psychometric properties of the FSFI. Despite these limitations, the authors believe that the FSFI-H is a valid and reliable instrument that can be used in settings to measure sexual functioning in Hungarian women who are in relationships and have no serious diseases that could influence their sexual activity.

## Conclusion

In our current study, we successfully translated and adapted the FSFI questionnaire into the Hungarian language (FSFI-H can be found in the Appendix).

## Appendix

### FSFI-H

#### Női szexuális funkció index kérdőív

(Hungarian Version of Female Sexual Function Index, FSFI-H)

*Kérjük, válaszoljon a következő kérdésekre, olyan egyértelműen amennyire csak lehetséges. Válaszait teljes mértékben bizalmasan kezeljük!*

*A lent található kérdések az elmúlt 4 hetes időszakra vonatkoznak. Kérjük, hogy egy kérdésre egy választ adjon meg.*

E kérdések megválaszolásakor a következő fogalom meghatározások érvényesek:

- A szexuális tevékenység magába foglalja a simogatást, előjátékot, maszturbációt és hüvelyi kapcsolatot.
- A szexuális közösülés: a férfi nemi szerv hüvelyi behatolása.
- A szexuális stimuláció magában foglalja a partnerrel való előjátékot, az önstimulációt (maszturbáció) vagy a szexuális fantáziát.
- A szexuális vágy vagy érdeklődés olyan érzés, amely magában foglalja azt, hogy szexuális élményt szeretne, fogékony a partner szexuális kezdeményezésére, gondolkodik vagy fantáziál a szexről.
- A szexuális izgalom olyan érzés, amely magában foglalja a szexuális izgalom fizikai és szellemi aspektusait, a nemi szervek melegségének vagy bizsergésének érzését, a nedvesedést, esetleg izom összehúzódásokat.
